# Association between HMGB1 and COPD: A Systematic Review

**DOI:** 10.1155/2015/164913

**Published:** 2015-12-21

**Authors:** Sebastiano Gangemi, Marco Casciaro, Giovanni Trapani, Sebastiano Quartuccio, Michele Navarra, Giovanni Pioggia, Egidio Imbalzano

**Affiliations:** ^1^School and Operative Unit of Allergy and Clinical Immunology, Department of Clinical and Experimental Medicine, University Hospital “G. Martino”, University of Messina, 98125 Messina, Italy; ^2^Department of Clinical and Experimental Medicine, University of Messina, 98125 Messina, Italy; ^3^Department of Chemical, Biological, Pharmaceutical and Environmental Sciences, University of Messina, University Pole Annunziata, 98168 Messina, Italy; ^4^Institute of Applied Sciences and Intelligent Systems (ISASI), Messina Unit, 98100 Messina, Italy

## Abstract

HMGB1 is an alarmin, a protein that warns and activates inflammation. Chronic obstructive pulmonary disease (COPD) is characterised by a progressive airflow obstruction and airway inflammation. Current anti-inflammatory therapies are poorly effective in maintaining lung function and symptoms of COPD. This underlines the need for finding new molecular targets involved in disease pathogenesis in order to block pathology progression. This review aims to analyse latest advances on HMGB1 role, utilisation, and potential application in COPD. To this purpose we reviewed experimental studies that investigated this alarmin as marker as well as a potential treatment in chronic obstructive pulmonary disease. This systematic review was conducted according to PRISMA guidelines. In almost all the studies, it emerged that HMGB1 levels are augmented in smokers and in patients affected by COPD. It emerged that cigarette smoking, the most well-known causative factor of COPD, induces neutrophils death and necrosis. The necrosis of neutrophil cells leads to HMGB1 release, which recruits other neutrophils in a self-maintaining process. According to the results reported in the paper both inhibiting HMGB1 and its receptor (RAGE) and blocking neutrophils necrosis (inducted by cigarette smoking) could be the aim for further studies.

## 1. Introduction

The high mobility group box (HMGB) protein family is the most represented protein family among the high mobility group proteins [1]. The HMGB are highly conserved and have four members (HMGB1–4). HMGB are types of protein motifs that are involved in DNA-binding [2, 3]. HMGB1 (also HMG1; HMG-1; HMG 1; amphoterin; p30) is the most highly expressed of all the HMG family members [4]. HMGB in addition to their nuclear functions have extracellular activity; that is, HMGB1 belongs to the “Damage Associated Molecular Pattern Molecules” (DAMPs) [5, 6]. DAMPs also known as alarmins are danger signals, which are released from damaged sort necrotic cells and “alert” the immune system by the activation of the “inflammasome” through the pattern recognition receptors (PRRs) found on the plasma membrane, inside endosomes after endocytosis, and in the cytosol (i.e., toll-like receptors), advanced glycosylation end product-specific receptor (RAGE), RIG-I-like receptors, NOD1-like receptors, and AIM2-like receptors [7, 8]. Nuclear HMGB1 is involved in many DNA activities such as DNA replication, repair, recombination, and transcription and in genomic stability. It also plays a significant role outside the cell in inflammation and immunity, as a mediator for cell growth, proliferation, and death [9, 10].

Chronic obstructive pulmonary disease (COPD) is characterised by a progressive airflow obstruction, airway inflammation, and systemic effects or comorbidities. Ageing itself and cigarette smoking are the most studied risk factors but not the only causative agents. COPD is the result of a gene-environment interaction. Infections and chronic inflammation, together with oxidative stress, are involved in the etiopathogenesis of the disease [11].

Current anti-inflammatory therapies are poorly effective in maintaining lung function and symptoms of COPD. This underlines the need for finding new molecular targets involved in disease pathogenesis in order to block pathology progression [11, 12].

This review aims to analyse latest advances on HMGB1 role, utilisation, and potential application in COPD. For this purpose, we reviewed experimental studies that investigated this alarmin as marker as well as a potential treatment in chronic obstructive pulmonary disease.

## 2. Methods

### 2.1. Research Strategy

This systematic review has been conducted according to PRISMA guidelines [13] employing two databases: PubMed and ScienceDirect. On these websites we searched for articles from January 1, 2005, to October 2015 using key terms related to COPD: “COPD,” “emphysema,” “bronchitis,” and “smoke” and one key term related to HMGB1: “HMGB1.” The electronic search strategy applied for PubMed is reported as an example in Table 1.

We decided to use as a rule of thumb the fact that the abstracts of those articles whose titles indicated they might have examined the association between COPD and HMGB1 were to be read. The entire article was read if the abstract indicated the article potentially met the inclusion criteria. Lastly we reviewed and searched references of the selected articles and the ones whose titles suggested that they could have researched the association between COPD and HMGB1 in order to identify additional studies that met the inclusion criteria.

### 2.2. Study Selection

Articles were included in the following review according to the following inclusion criteria: English language, publication in peer reviewed journals, and year of publication at least 2005. Articles were excluded by title, abstract, or full text for irrelevance to the topic in question. Further exclusion criteria were review articles and editorial comments. Furthermore, we arbitrarily decided to start our research from 2005 to give a more recent view of COPD findings.

### 2.3. Data Extraction

Three authors (Marco Casciaro, Sebastiano Quartuccio, and Giovanni Trapani) performed the initial search and independently reviewed and selected the references based on the inclusion and exclusion criteria.

Data derived from our research of articles includes study author names, publication dates, study designs (i.e., case-control, cross-sectional, and longitudinal), groups studied, clinical and biological variables, and outcome of interest of the study.

Principal outcome of interest included studies about advanced molecular targets on animals and humans as either disease marker or pathogenic mechanisms.

Given considerable heterogeneity in the study designs and subjects of the selected studies (in terms of biological and clinical variables), characteristics of the studied populations and protocols are summarised and the study outcome is reported using descriptive statistics without conducting any meta-analyses.

## 3. Results

**Figure 1:**
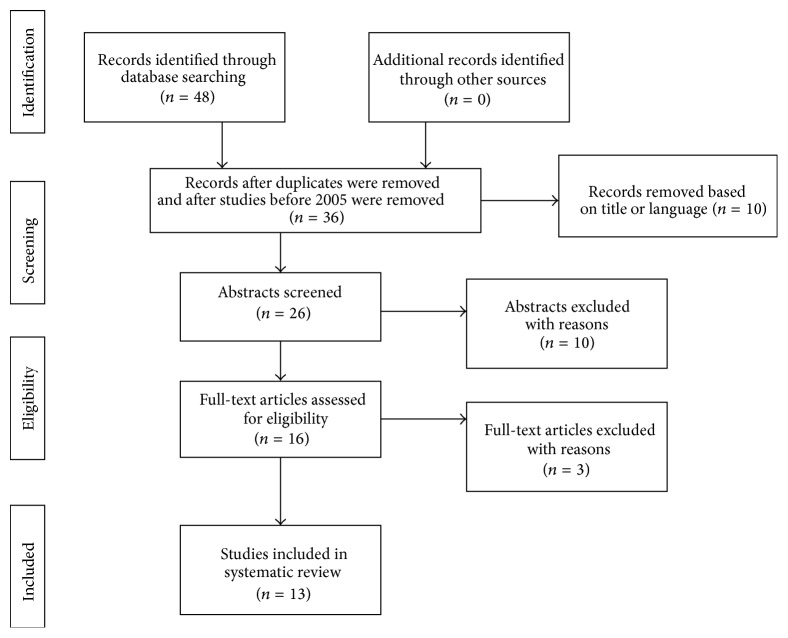
Flowchart of the results of the literature search.

In Figure 1 the flow of articles retrieved for the review is reported. As summarised in Table 2 a total of 13 studies assessing the association between HMGB1 and COPD were identified. As mentioned in Methods we decided to include only experimental studies since 2005 to provide a more recent perspective of HMGB1 in COPD.

According to Table 2, Shang et al. were the first in noticing the augmentation of plasmatic HMGB1 in COPD patients. In fact, they collected serum from a total of 271 patients (145 patients with non-small-cell lung cancer, 77 patients with COPD, and 49 controls patients). Serum HMGB1 levels in patients with lung cancer were significantly higher than those in COPD patients and healthy patients. But what emerged was also a difference between COPD patients and controls. Serum HMGB1 levels in patients with COPD were higher than healthy subjects [14].

These results pushed more studies about this possible association. In 2010 Ferhani et al. while looking whether or not it was possible to use HMGB1 as an inflammation marker analysed bronchoalveolar lavage (BAL) of 20 never-smoker patients, 20 smokers, and 30 smokers with COPD. The immunolocalization of HMGB1 and RAGE was assessed on bronchial biopsy and lung tissue sections. BAL levels of HMGB1 were comparable in never-smoker healthy subjects and in smokers without COPD but these levels were augmented in smokers with COPD. There was also a significant correlation between the individual BAL levels of HMGB1 and values of functional respiratory tests.

BAL levels of HMGB1 were also related positively to TNF-RII and IL-1b, and they were negatively related to IL-1RA with no correlation with TNF-*α*. HMGB1-positive cells were found in high concentration in bronchial mucosa of smokers with COPD, more often than in healthy smokers. Finally, Ferhani et al. found that RAGE was overexpressed in the airway epithelium and in smooth muscle of COPD patients and that it colocalized with HMGB1 [15].

Hou et al. selected 61 asthmatic patients, 47 COPD patients, and 34 healthy people. They decided to evaluate HMGB1 values both in sputum and in plasma. Compared with controls, HMGB1 levels in induced sputum were higher in asthmatic patients and in COPD patients. Plasma HMGB1 levels were similar to the ones obtained in sputum for exception of patients with mild asthma, in which levels were normal.

Moreover, patients with COPD showed a higher concentrations of sputum HMGB1 than patients with all severities of asthma. HMGB1 levels in plasma and in induced sputum in all subjects showed a negative correlation with lung function parameters. One more piece of data was that HMGB1 levels were positively correlated with neutrophil [16].

Bezerra et al. conducted a study in which they exposed mice to long-term cigarette smoking developing emphysema. After the spread of the chronic disease they analysed mice BAL noticing higher levels of alveolar macrophages and neutrophils than in control mice. In particular, MMP-12 and HMGB-1 were detected at high levels in alveolar macrophages from CS-exposed animals [17].

Kanazawa et al. performed TC, functional respiratory tests, and BAL on 14 nonsmokers subjects, on 13 smokers without COPD, and on 30 smokers with COPD. The analysis showed that HMGB1 levels from BAL performed on central airways did not significantly differ among the three groups; instead those levels from BAL obtained from peripheral airways were significantly higher in COPD patients than in nonsmokers or smokers without COPD.

In COPD patients there was no correlation between HMGB1 level in central airways and indexes of pulmonary function. On the contrary, HMGB1 level in peripheral airways was related to the functional tests.

Finally, what emerged from high resolution TC was a low-attenuation area significantly correlated with HMGB1 level in peripheral airways [18].

Wang et al. investigated how nuclear factor-*κ*B (NF-*κ*B) affected HMGB1 expression in an animal model of COPD. They administered a NF-*κ*B inhibitor to 48 rats (normal control, COPD rats, and COPD rats complicated with hypoxia). They reported an augmented level of HMGB1 mRNA and NF-*κ*B expression in lung tissue of rats affected by COPD. HMGB1 mRNA and protein expression were positively correlated with NF-*κ*B protein expression. After administration of the NF-*κ*B inhibitor, HMGB1 mRNA and protein expression in lung tissues were significantly reduced [19].

Ko et al. enrolled twenty-eight patients with pulmonary neoplasia. Eleven of them were smokers with COPD, eight smokers without COPD, and nine nonsmokers without COPD. HMGB1 was dosed on lung bioptic tissue and in serum. Data obtained revealed a negative correlation with lung function parameters in smoker subjects. HMGB1 expression was augmented in submucosa cells, in epithelium cell, and in alveolar cells in smoker subjects with COPD. None of these augmented expressions were confirmed in smokers without COPD and in healthy subjects [20].

Zhang et al. recruited 44 patients affected by COPD. They measured plasma levels of HMGB1, sRAGE, fibrinogen, and serum levels of high-sensitivity C-reactive protein while patients had an acute exacerbation of COPD. They observed that there was a decline of HMGB1, sRAGE, and hsCRP from the acute phase to the convalescence phase. HMGB1 and sRAGE levels were related to disease severity. Furthermore, HMGB1 and sRage levels were higher in smoker COPD patients compared with nonsmokers with COPD and exsmokers with COPD, respectively [21].

Zabini et al. investigated whether levels of HMGB1 were significantly higher in both patients with idiopathic pulmonary arterial hypertension (IPAH) (*n* = 14) and COPD pulmonary hypertension (*n* = 14). HMGB1 levels were augmented in both situations compared with healthy subjects. Bioptic lung tissue revealed the presence of HMGB1-positive cells surrounding remodelled vessels both in COPD with pulmonary hypertension and in IPAH lung samples. Moreover, they exposed these cells to progressively higher doses of HMGB1 in vitro, demonstrating HMGB1 proliferation effects on pulmonary arterial smooth muscle cells and human arterial endothelial cells [22].

Di Stefano et al. performed a study on 55 subjects. 32 patients were affected by a stable COPD, 12 patients were smokers with normal lung function, and 11 were nonsmokers with normal lung function. BAL concentration of HMGB1 was significantly diminished in patients with stable COPD compared with control healthy subjects (smokers and nonsmokers). However, after matching patients for age and smoking history this difference was lost [23].

Iwamoto and his group conducted a study, of the duration of 4 years, on 3 groups of patients (32 nonsmokers, 212 smokers without COPD, and 51 smokers with COPD). They found no difference between plasma HMGB1 levels among the different groups. In addition, there were no correlation between plasma HMGB1 levels and spirometric measurements. The only relevant data was the baseline plasma sRAGE lower concentration in smokers with and without COPD versus nonsmoker subjects. The lower plasma concentration of sRAGE was also associated with worse functional respiratory tests [24].

Starting from the well-known cigarette smoking (CS) causative effect on COPD development, Heijink et al. performed a study trying to demonstrate whether or not CS led to the release of DAMPs (i.e., HMGB1) which are known to amplify innate immune response. An in vivo-in vitro analysis of human BAL neutrophilic concentration was conducted together with an animal lung tissue and sputum dosage. Data confirmed that CS induced neutrophils necrosis with a subsequent release of DAMPs [25].

Pouwels et al. conducted a post hoc analysis of HMGB1 levels in serum of 40 COPD patients collected both during an exacerbation and during the stable phase. The same analysis was performed on the inducted sputum of almost the same patients (*n* = 35). No significative differences were found in sputum levels. On the contrary, serum levels of HMGB1 during COPD exacerbations were augmented. They also noticed a gender difference, with higher serum levels of HMGB1 in women during the acute phase [8].

## 4. Discussion

Intracellular HMGB binds to the minor groove of DNA, stabilizing nucleosome formation and regulating gene transcription and the activity of steroid hormone receptors. On the other hand, HMGB1 extracellularly has been demonstrated to have a role in inflammatory processes, neural outgrowth, smooth muscle cell chemotaxis, migration and proliferation of mesoangioblast cells, and cell proliferation in tumor and metastasis [26–28]. Also other authors confirmed that HMGB1 mediated cell transformation, growth, and resistance in neoplastic cells [29–31]. Extracellular HMGB1 functions are mediated via binding to specific membrane receptors including the receptor for advanced glycation end products (RAGE). RAGE activation gives origin to an intracellular cascade leading to the activation of the nuclear factor-*κ*B (NF-*κ*B) pathway and also of mitogen activated protein kinase (MAPK) and type IV collagenase (MMP-2/-9) which are fundamental for cancer cell characteristic [26, 29, 32]. It has been demonstrated that the blocking of the RAGE-HMGB1 axis suppresses tumor growth and metastasis [29, 30, 33].

DAMPs, including HMGB1, are released either by a damaged or by a necrotic cell and actively by the immune system. It has been seen that HMGB1 sustains the inflammatory process and promotes the proliferation of epithelial and mesenchymal cells (i.e., smooth muscle cells). These data support the hypothesis that HMGB1 could contribute to the pathogenesis of many diseases [34–38].

Due to these data Shang et al. focused their attention both on neoplastic and chronic inflammatory diseases by evaluating patients with lung cancer and patients with COPD. They first demonstrated that serum HMGB1 levels were significantly augmented both in patients with lung cancer and in patients with COPD [14].

Because of HMGB1 high affinity to RAGE and their intimate link, some studies about this molecule demonstrated that RAGE is present at very low concentration in many healthy tissues and that it is upregulated by high levels of HMGB1. Further studies revealed that, on the contrary, RAGE is expressed at high concentration in normal lung tissue suggesting a role in regulating lung homeostasis [39–41].

Ferhani et al. confirmed data previously obtained by demonstrating that smokers with COPD had higher levels of HMGB1 in their BAL fluid. In the same study they determined that macrophages, bronchial epithelial cells, and airway smooth muscle cells (ASM) of smoker patients had a high expression of HMGB1 and RAGE. These cells appear to represent a potential source of the secreted soluble form of HMGB1 in the airways [15].

With HMGB1-RAGE being overexpressed in all these cells, the hypothesis that lung tissue remodelling, typical in COPD, could be a consequence of this overexpression was advanced [15].

HMGB1-positive cells surrounding remodelled vessels of patients affected by COPD with secondary pulmonary hypertension were reported by Zabini et al. Data obtained suggest a proliferation effect on pulmonary arterial smooth muscle cells [22].

According to these findings and hypothesis, another study evaluated sputum and plasma concentrations of HMGB1 in patients affected by asthma and COPD. High concentration of the alarmin was found in both biological fluids of these patients. A neutrophil count positive correlation emerged [16]. Previously, Ito et al. reported HMGB1 secretion by neutrophils [42] and Rowe et al. demonstrated that HMBG1 could stimulate neutrophils chemotaxis [43]. On this basis Hou et al. postulated the theory of a positive feedback loop between HMGB1 and neutrophils, sustaining in ammation [16].

Bezerra et al. observed higher levels of neutrophil and macrophage cells in BAL of a mice model of COPD [17].

Data obtained by Heijink et al. confirmed that CS induces neutrophils necrosis with a subsequent release of DAMPs [25].

As mentioned before HMGB1 interaction with RAGE activates NF-*κ*B pathway sustaining inflammation; Wang et al. tried to target NF-*κ*B with an inhibitor on mice with COPD obtaining a downregulation of HMGB1 in lung tissue adding more elements in a blockage strategy of inflammation in an early phase of COPD [19].

In another study, BAL analysis of peripheral airways showed significantly higher levels of HMGB1 in COPD patients with positive correlation with functional respiratory tests [18]. Ko et al. reported that HMGB1 expression was augmented also in plasma, epithelium, submucosa, and alveolar cells in patients with COPD with a negative correlation with pulmonary tests [20].

Pouwels et al. and Zhang et al. studies both con rmed increased levels of serum HMGB1 in acute exacerbation of COPD [8, 21]. According to Zhang et al. there were also higher sRAGE levels than in controls [21].

The only study that, after delating possible outliers among patients, revealed no difference between BAL HMGB1 levels in COPD patients, smokers, and healthy subjects was the one made by Di Stefano et al. [23].

A more recent research emerged in order to evaluate the pathophysiological role of high mobility group box 1 and in particular its involvement in COPD remodelling. In this study Ojo et al. inhibited RAGE and TLR4 signalling. This blockage attenuated HMGB1 ability to increase epithelial cell reparation. These findings seem to better explain the role for HMGB1 in airway remodelling [44].

For a better understanding of HMB1 role in the development of chronic inflammatory diseases and about inflammatory stimuli leading to COPD, another recent study focused on the potential therapeutic effect of probiotics on cells exposed to cigarette smoking (CS). Mortaz et al. stimulated human macrophage with CS extract in the presence and absence of* L*.* rhamnosus* and* B*.* breve*. What emerged was a minor ability of CS to induce HMGB1 release in these cells. This data may open a new scenario on probiotics as anti-inflammatory agents in the prevention of diseases caused by a chronic inflammation stimulus such as CS [45].

Our group decided to focus on the role of HMGB1 in COPD and more specifically on giving a better comprehension about its pathophysiological role in the development of the disease. Our objective was to propose, while possible, new therapeutic strategies in facing the irreversible damage and subsequent substitution of healthy lung tissue with repair tissue, associated with a chronic inflammatory stimulus (i.e., cigarette smoking) on lungs themselves.

Almost every study confirmed an augmentation of either plasmatic or lung tissue levels of HMGB1 in patients affected by COPD [8, 14–22]. These levels were higher during the acute phase of COPD [8, 21] and in patients with pulmonary neoplasia [14].

Analysing cited literature, it emerges that the involvement of the HMGB1-RAGE axis is fundamental in the inflammatory-regenerative process. The interaction of HMGB1 with NF-*κ*B appears to be equally important; once these processes escape from physiological control they could determine neoplastic degeneration [27–31].

**Figure 2:**
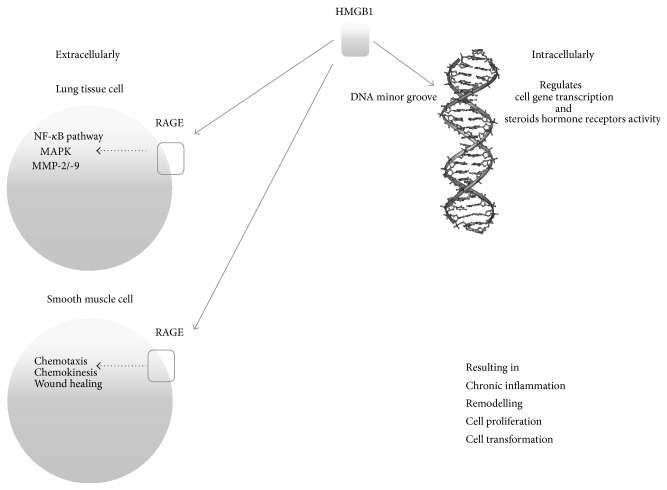
Intracellular and extracellular HMGB1 main interactions.

So it seems that HMGB1 level correlates with lung tissue damage and with the incipit of reparation processes. On the other hand, the level of this alarmin diminishes in a postacute phase. As represented in Figure 2, in a potential therapeutic key, blocking the binding of HMGB1 to the DNA minor groove may help in controlling the subsequent intracellular mechanism leading to gene transcription. In Figure 2 another potential therapeutic target is also shown; in fact, the extracellular interaction of HMGB1 with its receptor (RAGE) has been demonstrated, together with the above cited intracellular interaction, having a role in cell outgrowth. Interfering with these links may avoid permanent lung tissue damage and lung cells neoplastic transformation.

In conclusion in almost all the studies the fact that HMGB1 levels are augmented in smokers and in patients affected by COPD emerged. By the years it emerged that HMGB1 is an alarmin, a protein that warns and activates inflammation.

The studies reviewed let us to a better comprehension on how this alarmin is involved in the development of COPD. According to these studies it emerged that cigarette smoking, the most well-known causative factor of COPD, induces neutrophils death and necrosis. The necrosis of neutrophil cells leads to DAMPs release, in particular HMGB1, which recruits other neutrophils in a self-maintaining process. It emerged also that DAMPs could activate the inflammatory process by the interaction with the RAGE and worsen COPD exacerbation. In addition it was reported that blocking the NF-*κ*B led to an underexpression of HMGB1 in lung tissue.

Given these premises, both inhibiting HMGB1-RAGE axis and blocking neutrophils necrosis should be the aim of future further studies.

## Figures and Tables

**Table 1:** List of search terms entered into the PubMed search engines for identification the studies for this systematic review.

Number	Search term

1	COPD [all fields]
2	EMPHYSEMA [all fields]
3	BRONCHITIS [all fields]
4	SMOKE [all fields]
5	HMGB1
6	1 AND 5
7	2 AND 5
8	3 AND 5
9	4 AND 5
10	English [language]
11	2005 to present [publication date]

**Table 2:** Studies evaluating HMGB1 in the pathogenesis of COPD.

Authors	Manuscript title	Year	Human	Animals	Tissue	HMGB1 level in COPD patients	Laboratory tests	Functional or imaging tests
Shang et al. [14].	“Serum High Mobility Group Box Protein 1 as a Clinical Marker for Non-Small-Cell Lung Cancer”	2009	x	—	Serum	>	—	Spirometry

Ferhani et al. [15].	“Expression of High-Mobility Group Box 1 and of Receptor for Advanced Glycation End Products in Chronic Obstructive Pulmonary Disease”	2010	x	—	BAL Lung T.	> >	TNF-*α*, TNF-RII, IL1-*β*, IL1-RA, RAGE	Spirometry

Hou et al. [16].	“High Mobility Group Protein B1 (HMGB1) in Asthma: Comparison of Patients with Chronic Obstructive Pulmonary Disease and Healthy Controls”	2011	x	—	Sputum Plasma	> >	—	Spirometry

Bezerra et al. [17]	“Long-Term Exposure to Cigarette Smoke Impairs Lung Function and Increases HMGB-1 Expression in Mice”	2011		x	BAL Lung T.	> >	SOD, CAT, GPx, MMP-2,9,12	—

Kanazawa et al. [18]	“Validity of HMGB1 Measurement in Epithelial Lining Fluid in Patients with COPD”	2012	x	—	Central BAL Peripheral BAL	= >	IL-8, PMN elastase	TC Spirometry

Wang et al. [19]	“Effect of NF*‐κ*B Inhibitor on High-Mobility Group Protein B1 Expression in a COPD Rat Model”	2013	—	x	Lung T.	>	NF-*κ*B	—

Ko et al. [20]	“High Expression of High-Mobility Group Box 1 in the Blood and Lungs Is Associated with the Development of Chronic Obstructive Pulmonary Disease in Smokers”	2014	x	—	Plasma Biopsy	> >	—	Spirometry

Zhang et al. [21]	“Changes of HMGB1 and sRAGE during the Recovery of COPD Exacerbation”	2014	x	—	Plasma	>	Fibrinogen, hsCRP, RAGE	Spirometry

Zabini et al. [22]	“High-Mobility Group Box-1 Induces Vascular Remodelling Processes via c-Jun Activation”	2015	x	—	Lung T.	>	TLR4, RAGE	—

Di Stefano et al. [23]	“Innate Immunity but Not NLRP3 Inflammasome Activation Correlates with Severity of Stable COPD”	2014	x	—	BAL	=	IL-27, IL-37, NRLP7	—

Iwamoto et al. [24]	“Soluble Receptor for Advanced Glycation End-Products and Progression of Airway Disease”	2014	X	—	Plasma	=	sRAGE	—

Heijink et al. [25]	“Cigarette Smoke-Induced Damage-Associated Molecular Pattern Release from Necrotic Neutrophils Triggers Proinflammatory Mediator Release”	2015	x	x	BAL Sputum	—	—	—

Pouwels et al. [8]	“Increased Serum Levels of LL37, HMGB1 and S100A9 during Exacerbation in COPD Patients”	2015	x	—	Serum Sputum	> =	Galectin-3, S100A9, sRAGE, LL37, dsDNA	—

Lung T.: bioptic lung tissue.
